# On-The-Go Hyperspectral Imaging Under Field Conditions and Machine Learning for the Classification of Grapevine Varieties

**DOI:** 10.3389/fpls.2018.01102

**Published:** 2018-07-25

**Authors:** Salvador Gutiérrez, Juan Fernández-Novales, Maria P. Diago, Javier Tardaguila

**Affiliations:** Instituto de Ciencias de la Vid y del Vino - University of La Rioja, CSIC and Gobierno de La Rioja, Logroño, Spain

**Keywords:** MLP, plant phenotyping, discrimination, sensors, proximal sensing, remote sensing, non-invasive sensors

## Abstract

Grapevine varietal classification is an important plant phenotyping issue for grape growing and wine industry. This task has been achieved from destructive techniques like classic ampelography and DNA analysis under laboratory conditions. This work displays a new approach for the classification of a high number of grapevine (*Vitis vinifera* L.) varieties under field conditions using on-the-go hyperspectral imaging and different machine learning algorithms. On-the-go imaging was performed under natural illumination using a hyperspectral camera mounted on an all-terrain vehicle at 5 km/h. Spectra were acquired over two different leaf phenological stages on the canopy of 30 different varieties on a commercial vineyard located in La Rioja, Spain. A total of 1,200 spectral samples were generated. Support vector machines (SVM) and artificial neural networks (multilayer perceptrons, MLP) were used for the development of a large number of models, testing different algorithm parameters and spectral pre-processing techniques. Both classifiers yielded notable performance values and were able to train models with recall F1 scores and area under the receiver operating characteristic curve marks up to 0.99 for 5-fold cross validation. Statistical analyses supported that the best SVM kernel was linear and the best activation function for MLP was the hyperbolic tangent function. The prediction performance for individual varieties of MLP ranged from 0.94 to 0.99, displaying low levels of variability. In the case of SVM, slightly higher differences were obtained, ranging from 0.83 to 0.97 for individual varieties. These results support the possibility of deploying an on-the-go hyperspectral imaging system in the field capable of successfully classifying leaves from different grapevine varieties. This technology could thus be considered as a new useful non-destructive tool for plant phenotyping under field conditions.

## 1. Introduction

Plant phenotyping address the description of the plant's anatomical, physiological and biochemical properties (Walter et al., 2015). As grapevine growing and wine industry have a high economical and social impact, the interest of plant phenotyping is increasing in this context. In practice, however, phenotypes from controlled conditions rarely agree with those in field environments (Nelissen et al., 2014; Poorter et al., 2016). For this reason, in field plant phenotyping has become a necessity, but it still remains as a difficult task. The development of new technologies and methodologies for the precise phenotyping and monitoring of grapevines under field conditions would definitely improve grape quality (and, thus, wine quality), a key factor for the industry.

Grapevine variety is a key feature of final product in terms of price, cultivation, etc. (Clarke and Rand, 2015). In the world, there exist several thousands of grapevine varieties, and ampelography has been the classic approach for their identification (Galet, 1979). Ampelography aims at extracting morphological differences between the leaves and grape berries, but it has always required specialized human resources. This methodology has gradually made way to modern and more precise identification approaches, such as wet chemistry (Altube et al., 1991) or DNA analysis (Sefc et al., 2001; Borrego et al., 2002; Pelsy et al., 2010). Nevertheless, the difficulty to fast and easily apply these techniques and their destructive nature makes them unable to be translated to a real time in-field application.

The advances in the research and development of applied spectroscopy—which involves the interaction between radiation and matter at specific wavelengths—reveals this technology as a serious candidate to address the varietal classification goal. Likewise, many spectroscopic approaches have been developed toward this objective in several crops, such as barley malt (Porker et al., 2017), lotus seed (Guo et al., 2017b), pummelo (Li et al., 2016), or strawberry (Sánchez et al., 2012). Even works on in-field grapevine varietal classification using a near-infrared (NIR) device can be found in the literature (Gutiérrez et al., 2015, 2016). Hyperspectral imaging combines the potential of spectroscopy and the additional information that a two-dimensional space provides, and thus opens a new way to the development of spectroscopic methodologies. Particularly, hyperspectral images of grapevine leaves enable the development of varietal and clone classification models, as demonstrated by previous works (Diago et al., 2013; Fernandes et al., 2015). However, these studies worked with a very limited number of classes (no more than four), under laboratory conditions and required sample preparation. These pitfalls raise the necessity of taking a further step and deploying hyperspectral imaging directly in the field, opening a new frontier for the on-the-go classification of a large number of grapevine varieties, hence removing the requirements of laboratory conditions and even sample picking. This new application could be useful for commercial vineyards, nurseries, appellation boards, etc. Some authors have previously demonstrated the possibility of performing outdoor hyperspectral imaging in several crops (Underwood et al., 2017; Wendel and Underwood, 2017; Williams et al., 2017), and this bolsters the development of new on-the-go hyperspectral solutions for grapevine-related problems.

As exposed, hyperspectral imaging brings much richer data in relation to quantity and quality, but this feature also carries a big burden that needs to be handled: the huge amount of data that hyperspectral acquisitions implies. For this reason, efficient and intelligent data analysis is an almost compelled necessity. Machine learning provides numerous techniques for predictive applications by learning and forecasting data (Han et al., 2011; Witten et al., 2016), and it has been extensively used in innumerable fields. Two of the most reliable and adaptable algorithms for the development of supervised classification models are support vector machines (SVM) and artificial neural networks (ANN).

SVM are algorithms that are based on a *kernel* that translates the input data into higher dimensional spaces (Capparuccia et al., 1995). In these, SVM try to find hyperplanes that maximize the distance to the nearest point (projected in the new dimensional space) of any of the input classes. The adequate selection of a kernel is crucial when applying SVM to a problem, as specific kernels can fit better than other depending on the data modeled. SVM were originally conceived as binary classifiers, but multi-label classification SVM can be developed by splitting the original multi-class problem into several smaller binary classification ones using approaches as one-versus-all (training one model per class versus all the rest) or one-vs.-one (training one model per class for each one of the remaining classes). Applications based on SVM models can be widely found in plant science, like nitrogen evaluation (Gao et al., 2017), characterization of invasive grass distribution (Dronova et al., 2017) or seed development genetics (Ni et al., 2016). ANN are a popular machine learning approach extensively used for classification and regression purposes. Originally suggested by McCulloch and Pitts (1943), the modern concept of ANN was developed by Werbos (1974). ANNs try to emulate the behavior of a biological neural network, by deploying a net of basic interconnected units (neurons) and arranging them into a set of discrete layers (one-layer or multi-layer). In Rumelhart et al. (1986), *error backpropagation* feature was introduced, a process that finds the gradients of the neurons' weights to adjust them, from the last layer to the first one. ANNs can also be found in multiple applications for plant science, e.g., leaf area index calculation (Yuan et al., 2017), rootstock genetics (Arab et al., 2017) or disease detection (Pérez-Bueno et al., 2016). For this reason, a deep analysis of how these algorithms and their multiple parameter settings behave with hyperspectral data is desirable, as they arise as powerful tools for the varietal classification objective.

The objective of this study was to develop a new application for the classification of a large number of grapevine (*Vitis vinifera* L.) varieties using on-the-go hyperspectral imaging under field conditions and machine learning algorithms.

## 2. Materials and methods

On-the-go hyperspectral imaging was performed in a commercial vineyard on a moving vehicle under field conditions and natural light, at two different phenological stages in a given season. A large amount of parameter combinations for spectral pre-processing and machine learning classification models were tested and statistically analyzed to evaluate the influence of the different parameters and obtain the best configuration for the machine learning classifiers.

### 2.1. Experimental layout

The study was conducted in a 1.8 ha commercial vineyard located in Logroño, La Rioja, Spain (Lat. 42° 2″ 4.5″″, Long. -2° 30″ 49.6^′*′′′*^ Alt. 484 m), during two different days with clear weather corresponding to two different phenological stages of season 2017: 10 August—1 week post-veraison, at stage 36 of the modified Eichhorn and Lorenz system (Coombe, 1995)—and 11 October—1 week post-harvest, at stage 41. Grapevines (*Vitis vinifera* L.) were grafted on rootstock R-110 and trained to a vertically shoot-positioned trellis system. Plants were planted in 2001 with a Northwest-Southeast orientation at 3.00 × 1.20 m inter and intra row distances. Mechanical tillage was applied for vineyard soil management. Thirty different international grapevine varieties, uniformly irrigated across the season, were used in this study. From these, 16 white varieties were present: Baladí, Blanca Cayetana, Calagraño, Catalán Blanco, Chardonnay, Chenin Blanc, Cigüente, Palomino, Pardina, Parellada, Pedro Ximénez, Perruno Fino, Picapoll Blanco, Pinot Blanc, Sauvignon, Semillón. The other 14 were red varieties: Brancellao, Cabernet Franc, Cabernet Sauvignon, Calop Negro, Carnelian, Centurion, Concord, Crujidera, Pinot Noir, Rubired, Rufete, Sousón, Syrah, and Tempranillo. For each variety, 10 plants (along 12 m) were imaged. The 30 different varieties were randomly planted across the whole vineyard plot.

### 2.2. On-the-go hyperspectral imaging

**Figure 1:**
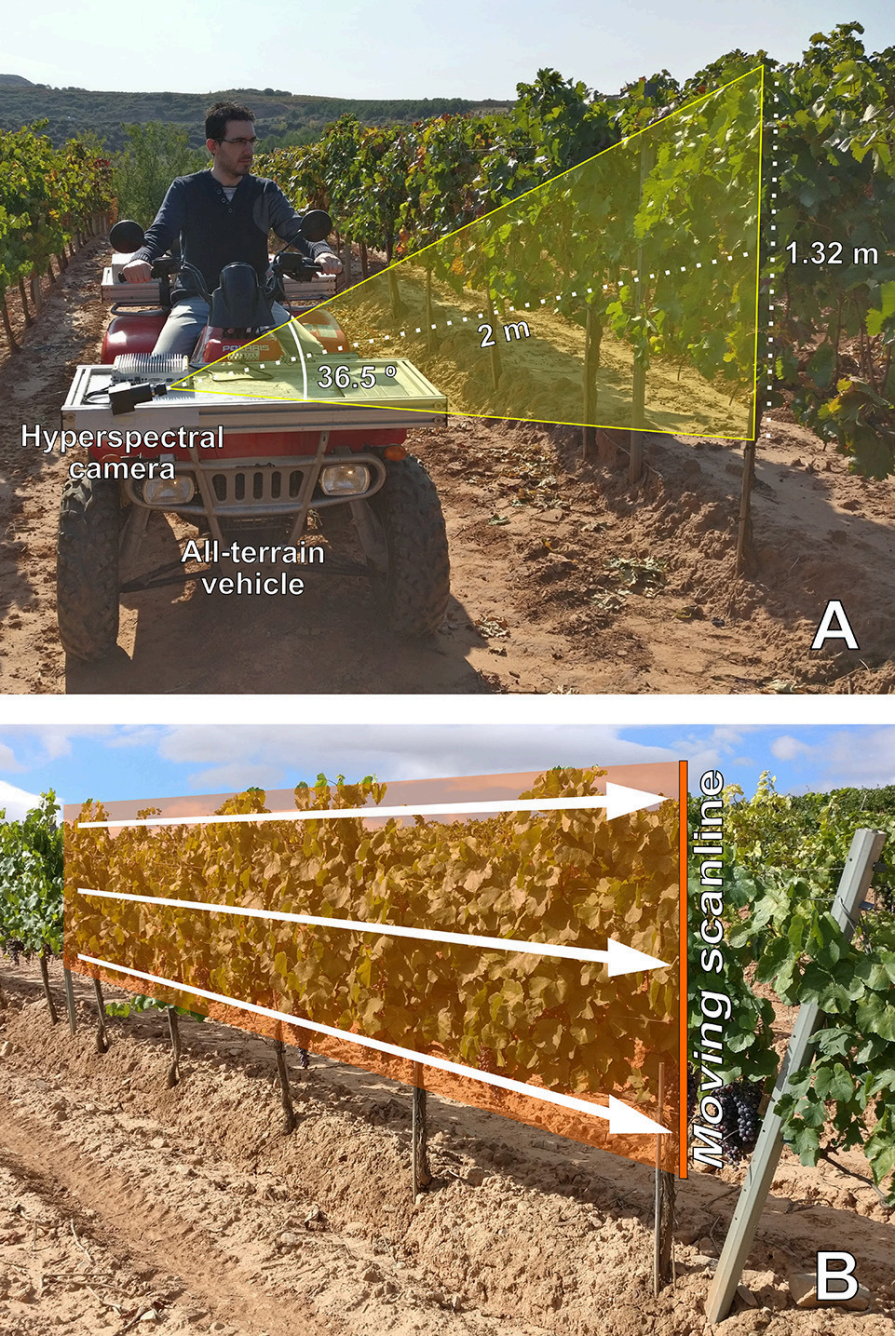
**(A)** On-the-go hyperspectral imaging on an all-terrain vehicle in a vertically shoot positioned vineyard located in Logroño, La Rioja (Spain). Spectral acquisition was performed on the sun-exposed canopy side at 5 km/h. (The authors declare that written and informed consent has been obtained from the depicted individual in this image, for the publication of this identifiable image). **(B)** Construction of a two-dimensional hyperspectral image by push broom. The camera's scanline, that was acquiring spectral information from a vertical line over the vineyard canopy, was moved by the motion of the all-terrain-vehicle. Thus, the composition of the image was performed by this scanline dragging at constant speed.

The on-the-go acquisition of hyperspectral images was performed using a Resonon Pika L VNIR hyperspectral imaging camera (Resonon, Inc., Bozeman, MA, USA) mounted on the front part of an all-terrain vehicle (ATV) (Trail Boss 330, Polaris Industries, MN, USA), on a lateral point of view at 2.0 m of distance (Figure 1A). The camera covered the spectral range from 400 to 1,000 nm, with a spectral resolution of 2.1 nm (300 bands) and a spatial resolution of 900 pixels. Using an objective lens with a focal length of 8 mm, the field of view (FOV) was 36.5°, and casted a vertical recording line covering 1.32 m of the northeast canopy side, only with the natural illumination from the sun (between 10:00 and 12:00).

The camera configuration was set up at 108 frames per second (FPS) with integration time of 6.53 ms, to maximize the trade-off between an acceptable image composition of the plants and spectral quality (avoiding signal saturation). In order to take into account the natural, variable illumination, at the beginning of the hyperspectral recording, for each variety, a Spectralon® white reference was manually presented to the camera and statically imaged. The dark current (that corresponds to inherent electronic noise) was measured with the camera lens covered. Afterwards, the 10 plants of that specific variety were measured at a constant speed of 5 km/h. The horizontal movement from the ATV composed the whole hyperspectral image by push broom scanning (Figure 1B). The plants from each varietal recording comprised an average of 1,800 scanlines (columns in the hyperspectral image), 900 pixels each column. Therefore, each varietal hyperspectral image was composed of, on average, 1,620,000 pixels (i.e., spectra).

All the raw information from the camera (acquired as light intensity) was translated into reflectance, using the following equation:

(1) $$\begin{array}{l} R\left({\bar{\text{d}}}_{r},\;\lambda \right)=\frac{G\left({\bar{\text{d}}}_{r},\;\lambda \right)-D\left({\bar{\text{d}}}_{r},\;\lambda \right)}{W\left({\bar{\text{d}}}_{r},\;\lambda \right)-D\left({\bar{\text{d}}}_{r},\;\lambda \right)} \end{array}$$

where ${\bar{\text{d}}}_{r}$ is a position, λ is a wavelength, *G* is the intensity of the light reflected by the target, *W* is the intensity of the light coming from the white reference, and *D* is the dark current. Afterwards, the absorbance (log 1/*R*) was calculated as the final unit to be used in computation. From this absorbance spectra, the first and last group of 25 bands were discarded to avoid the noise commonly present in both spectral signal's tails. Therefore, each spectrum comprised a total of 250 bands.

### 2.3. Building the datasets

From the raw hyperspectral images, a semi-automatic dataset building process programmed in Python 3.6.1 was performed in two steps: the segmentation and filtering of the leaf spectra, and the generation of the samples for each grapevine variety.

#### 2.3.1. Segmentation and filtering of leaf spectra

**Figure 2:**
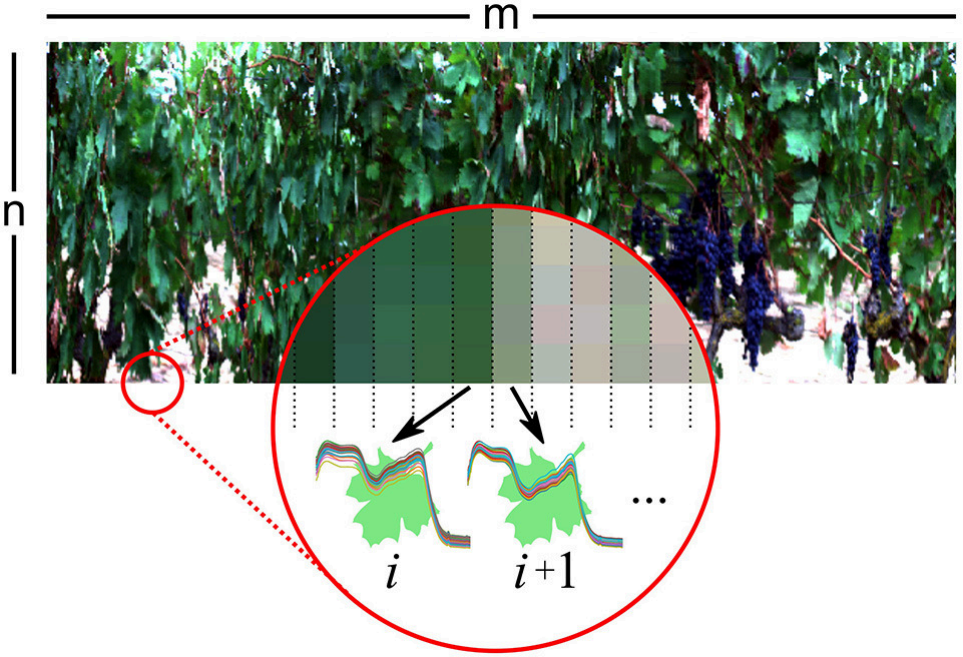
Each *m* × *n* hyperspectral image was processed column by column. For each column *i*, each pixel (spectrum) was compared with a signature leaf spectrum. If a certain threshold of belonging was surpassed, the pixel was marked as leaf pixel. Afterwards, all leaf pixels from the column *i* were averaged.

The following procedure was applied to each variety hyperspectral image. From the *n* × *m* image (where *m* is the number of columns and *n* the number of pixels in each column), one manually selected average leaf spectrum was extracted and used as signature spectrum (the pure reference spectrum of a leaf of that image). Afterwards, for each column, all the spectra corresponding to leaves were automatically selected and averaged as described:

A Saviztky-Golay smoothing and derivative (Savitzky and Golay, 1964) was applied to the leaf signature spectrum. Afterwards, for each column, each one of its pixels were picked and its spectrum in absorbance was extracted, applying the same Savitzky-Golay smoothing and derivative. Then, the correlation coefficient between the pixel spectrum and the signature spectrum was computed, and if the Pearson's *r* was greater than 0.90, the spectrum was therefore positively identified as a leaf's spectrum and added into a selected leaves set. After all the pixels in the column were tested, the average spectrum from the selected leaves set was computed and considered as the average spectrum from all the leaves in that column. Figure 2 represents a visual summary of this procedure.

After all the columns were processed, *m* mean spectra (one per column) were extracted for each variety altogether. On average, for all the varieties, the mean spectra was computed from 481 pixels (a 53.4% of the column pixels).

#### 2.3.2. Generation of the dataset samples

For each variety, the *m* mean leaf spectra were divided into 40 consecutive sets with a size of *m*/40 spectra. The average spectra from those sets were obtained and, thus, 40 leaf spectra per variety (four per plant) were finally generated, following previous methodologies by Gutiérrez et al. (2015, 2016). Having 30 varieties and two measurement days, a total of 2,400 samples (80 per variety) were generated, each one obtained from the averaging of approximately 21,500 spectra (86,000 leaf pixels per plant).

### 2.4. Spectral pre-processing and machine learning modeling

In the development of prediction models from spectral information, the raw absorbance data is seldom used directly as input. Spectral pre-processing is a common step that seeks to remove most of the noise that is inherent to many spectral acquisitions. As several algorithms and parameters can be applied, and they noticeably affect the spectral shape, the influence of two different pre-processing techniques were tested in the training of the varietal classification models:

- **Scatter correction**. Sometimes, it is usual for spectral signal to retain interferences of scatter. One of the techniques usually applied for this correction is the combination of standard normal variate (SNV) followed by a de-trending (Barnes et al., 1989; Dhanoa et al., 1995). Nevertheless, there are situations in which the application of scatter correction is not necessary, so for this study it was tested the use of SNV + de-trending and the complete omission of this scatter correction step.
- **Smoothing filtering**. Savitzky-Golay filtering along with a derivative function (Savitzky and Golay, 1964) is commonly used in spectroscopy, as they are able to remove noise from external sources and to emphasize certain parts from the original spectrum. The combination of two derivative orders (first and second) and three different Savitzky-Golay window sizes (5, 9, and 15) was tested.

Regarding machine learning modeling, two different classification algorithms were tested:

- **Support vector machines (SVM)**. SVMs are algorithms based on kernels that transform the original data into high-dimensional feature spaces (Capparuccia et al., 1995). The parameters tested for SVM were: the penalty parameter *C* (six different values: 0.01, 0.1, 1, 10, 100, and 1,000) and three different kernels (*linear, polynomial*, and *radial basis function*–*RBF*). A total of 18 parameter combinations were thus generated. As SVM are binary classification algorithms, a one-*vs*.-one approach was followed in this work to perform multi-class classification Bishop (2006). This approach trains *n*(*n* − 1)/2 binary models (where *n* is the number of classes), one for each one of the two-classes combinations that can be arranged. As in this case all the classes had the same number of samples, no bias was introduced in the models, hence avoiding over-estimation of a majority class.
- **Multilayer perceptrons (MLP)**. MLPs are a kind of artificial neural networks (ANN) that consist of at least three layers of neurons and make use of backpropagation in the training process (Hornik et al., 1989). The parameters tested for MLP were: number of neurons in the hidden layer (*t*: the sum of the number of attributes and classes. *a*: half the amount of *t*; *i*: the number of attributes), activation function for the hidden layer (*logistic*: logistic sigmoid function; *tanh*: hyperbolic tangent function; *relu*: rectified linear unit function) and using or not a warm start (reuse or reject previous solutions in the ANN training process). The total number of combinations were also 18.

**Figure 3:**
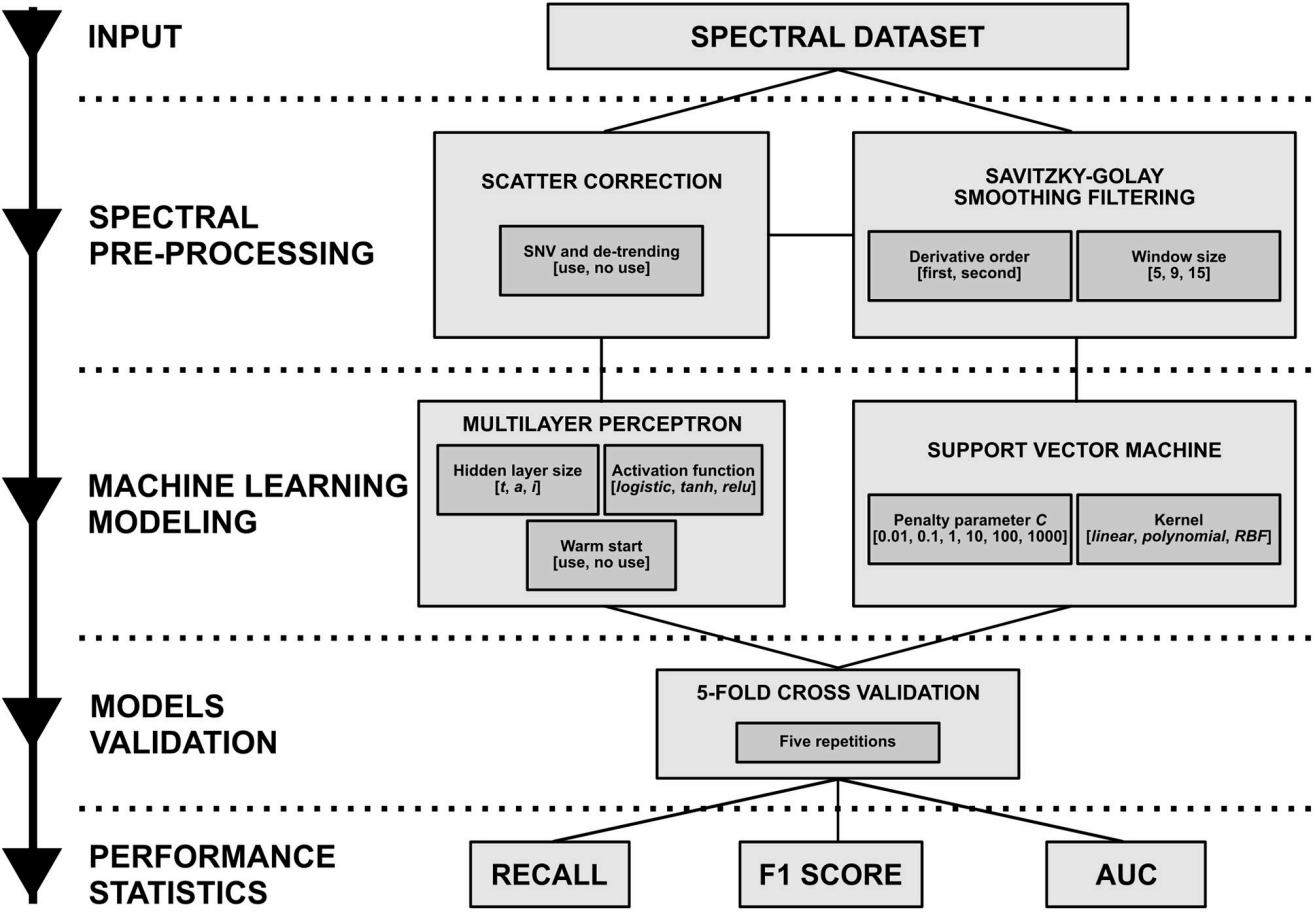
Experimental modeling diagram summarizing the analyses performed. From the spectral dataset (input), different combinations of various pre-processing techniques were applied, modeled using two machine learning algorithms (with many parameters) and validated by several 5-fold cross validation replicates. Finally, three performance statistics were evaluated.

Each developed model was evaluated using a stratified *k*-fold cross validation, with *k* = 5. In a *k*-fold cross validation, *k* models are trained with *k*−1 folds and tested with the remaining fold, rotating the latter until all of them have been used. The average performance of the *k* models is thus considered as the performance of the cross validation. Five replicates of 5-fold cross validation were also carried out, each one of them with random fold splits. In summary, having two options for scatter correction, six combinations for smoothing filtering, two algorithms, 18 parameter combinations for each one and five cross validation replicates, a total of 2,160 classification models were developed. The performance statistics used were the recall, F1 score, defined as:

(2) $$\begin{array}{l} \text{recall}=\frac{tp}{tp+fn}=\frac{\text{number of correctly classified samples}}{\text{total number of testing samples}} \end{array}$$

(3) $$\begin{array}{l} \text{F}1\text{ score}=2\times \frac{\text{precision }\times \text{ recall}}{\text{precision }+\text{ recall}} \end{array}$$

where *tp* is *true positives* (number of samples correctly classified) and *fn* (number of samples incorrectly classified) is *false negatives*, and the area under the receiver operating characteristic curve (AUC) (Bradley, 1997), computed from the SVM and ANN class membership probability estimates. The performance statistics used were averaged among all the classes. An experimental modeling diagram is presented in Figure 3.

The evaluation of the models was developed using Python 3.6.1 and scikit-learn 0.18.1. The training of the MLP was performed using on scikit-learn multilayer perceptron implementation (Pedregosa et al., 2011). Statistical tests were carried out using InfoStat software (Córdoba, Argentina), version 2017, using Tukey's range test at a significance level *p* = 0.05.

## 3. Results

### 3.1. Influence of scatter correction and derivative order

The comparison of means of classification recall for scatter correction was performed for each algorithm. No statistically significant differences were found between the means from any statistic when using and omitting SNV followed by a de-trending (data not shown). Therefore, the successive statistical analyses were performed without splitting by scatter correction treatments. Besides, the influence of the first and second order derivatives was analyzed, and statistically significant differences were found between them for MLP (*p* < 0.0001 for the three performance statistics) and SVM (mean recall with *p* < 0.05 and F1 with *p* < 0.01) toward the second order derivative.

### 3.2. Influence of smoothing filtering

The statistical analyses for the recall results attending to the different Savitzky-Golay window size are gathered in Table 1.

**Table 1:** Comparison of means of classification recall, F1 score and AUC for each Savitzky-Golay window size by algorithm and derivative order.

			**Window size**			
**Algorithm**	**Performance statistic**	**Derivative order**	**5**	**9**	**15**	**Significance**
SVM	Recall	First	0.8839	0.8648	0.8351	*n.s*.
		Second	0.9024	0.8947	0.8842	*n.s*.
	F1 score	First	0.8934	0.8747	0.8450	*n.s*.
		Second	0.9142	0.9058	0.8938	*n.s*.
	AUC	First	0.9309	0.9305	0.9297	*n.s*.
		Second	0.9339	0.9328	0.9265	*n.s*.
MLP	Recall	First	0.9796 **a**	0.9678 **b**	0.9404 **c**	^***^
		Second	0.9905 **a**	0.9842 **b**	0.9804 **c**	^***^
	F1 score	First	0.9796 **a**	0.9687 **b**	0.9404 **c**	^***^
		Second	0.9905 **a**	0.9842 **b**	0.9804 **c**	^***^
	AUC	First	0.9998 **a**	0.9995 **b**	0.9986 **c**	^***^
		Second	0.9999 **a**	0.9998 **b**	0.9996 **c**	^***^

*The values represent the average recall*.

*Dissimilar lowercase letters within rows represent statistically different means among different window sizes, using Tukey's range test at a significance level p = 0.05*.

*AUC, area under the receiver operating characteristic curve; SVM, support vector machine; MLP, multilayer perceptron. n.s., not significant (p≥0.05)*;

*** *p < 0.001*.

In all cases, the classification outcomes from the MLP surpassed those from the SVM models.

SVM results did not yield statistically significant differences between window size for both derivative orders, with values that ranged from 0.84 to 0.90 for recall, from 0.84 to 0.91 for F1 score and 0.93 in all cases for AUC. The best scores came from the second derivative smoothing with the lower window size values (five and nine), and in all cases the first derivative casted equal or lower recall outcomes.

MLP showed strong and consistent statistically significant differences, at *p* < 0.001 for both derivative orders, across all the performance statistics, supporting that the best scores were obtained in general using the second order derivatives (regardless the window size). In both first and second order derivatives, there existed a trend in which the lower the value of the window size, the better the recall values.

### 3.3. Analysis of the algorithm parameters

The results for the statistical analyses per parameter value are gathered in Tables 2, 3, for SVM and MLP respectively.

**Table 2:** Comparison of means of classification recall, F1 score and AUC for the different parameters tested for support vector machine (SVM).

**Parameter**	**Value**	**Average recall**	**Average F1 score**	**Average AUC**
Penalty parameter (*C*)	1,000	0.99 *a*	0.99 *a*	0.99 *a*
	100	0.99 *a*	0.99 *a*	0.99 *a*
	10	0.98 *a*	0.98 *a*	0.99 *a*
	1	0.92 *b*	0.94 *b*	0.99 *a*
	0.1	0.73 *c*	0.75 *c*	0.98 *a*
	0.01	0.65 *d*	0.68 *d*	0.60 *b*
	Significance	^***^	^***^	^***^
Kernel	*Linear*	0.99 *a*	0.99 *a*	0.99 *a*
	*Radial basis function*	0.90 *b*	0.90 *b*	0.95 *b*
	*Polynomial*	0.74 *c*	0.77 *c*	0.84 *c*
	Significance	^***^	^***^	^***^

*Dissimilar lowercase letters within the different parameter values represent statistically different means, using Tukey's range test at a significance level p = 0.05*.

*** *p < 0.001*.

**Table 3:** Comparison of means of classification recall, F1 score and AUC for the different parameters tested for multilayer perceptron (MLP).

**Parameter**	**Value**	**Average recall**	**Average F1 score**	**Average AUC**
Hidden layer	t	0.9746 *a*	0.9746 *ab*	0.9995 *ab*
	i	0.9757 *a*	0.9757 *a*	0.9996 *a*
	a	0.9717 *b*	0.9717 *b*	0.9994 *b*
	Significance	^**^	^**^	^**^
Activation function	*tanh*	0.9855 *a*	0.9855 *a*	0.9998 *a*
	*relu*	0.9837 *a*	0.9837 *a*	0.9998 *a*
	*Logistic*	0.9527 *b*	0.9527 *b*	0.9990 *b*
	Sign.	^***^	^***^	^***^
Warm start	True	0.9740	0.9739	0.9995
	False	0.9739	0.9739	0.9994
	Significance	*n.s*.	*n.s*.	*n.s*.

*Dissimilar lowercase letters within the different parameter values represent statistically different means, using Tukey's range test at a significance level p = 0.05*.

*n.s., not significant (p ≥ 0.05)*;

** *p < 0.01*.

*** *p < 0.001*.

*AUC, area under the receiver operating characteristic curve. t, the number of neurons is the sum of the number of attributes and classes. i, the number of neurons is the number of attributes. a, the number of neurons is half the amount of t. tanh, hyperbolic tangent function. relu, rectified linear unit function. logistic, logistic sigmoid function*.

The models trained with SVM presented large differences depending on the specific values selected (averages with high variability, from 0.65 up to 0.99 for recall, from 0.68 to 0.99 for F1 and from 0.60 to 0.99 for AUC), especially regarding the *C* parameter. In this case, a noticeable gap in terms of average recall can be found between *C* values equal or greater than one and 10 (that performed significantly better) and those that lied below that (whose scores casted worst results). For the different SVM kernel values, the three of them presented significant differences in all the statistics, being the linear *kernel* the one with the highest score. The *polynomial* kernel presented significantly lower average values.

The variability of the MLP results was considerably lower than that from SVM, with all values above the 0.95 mark and up to 0.99, for all the performance statistics. In terms of recall values, the number of neurons in the neural network models (hidden layer parameter) presented significant differences when selecting *i* or *t* over *a* (with slightly lower values for the latter), differences that were almost similar for both F1 and AUC. Nevertheless, the activation function responded differently depending on their selected values with the same behavior for the three statistics. In the first case, the *tanh* and *relu* functions worked significantly better than the *logistic* **one** (Table 3). The use of warm start exhibited no statistical significant differences in any case.

### 3.4. Prediction capability per variety

**Figure 4:**
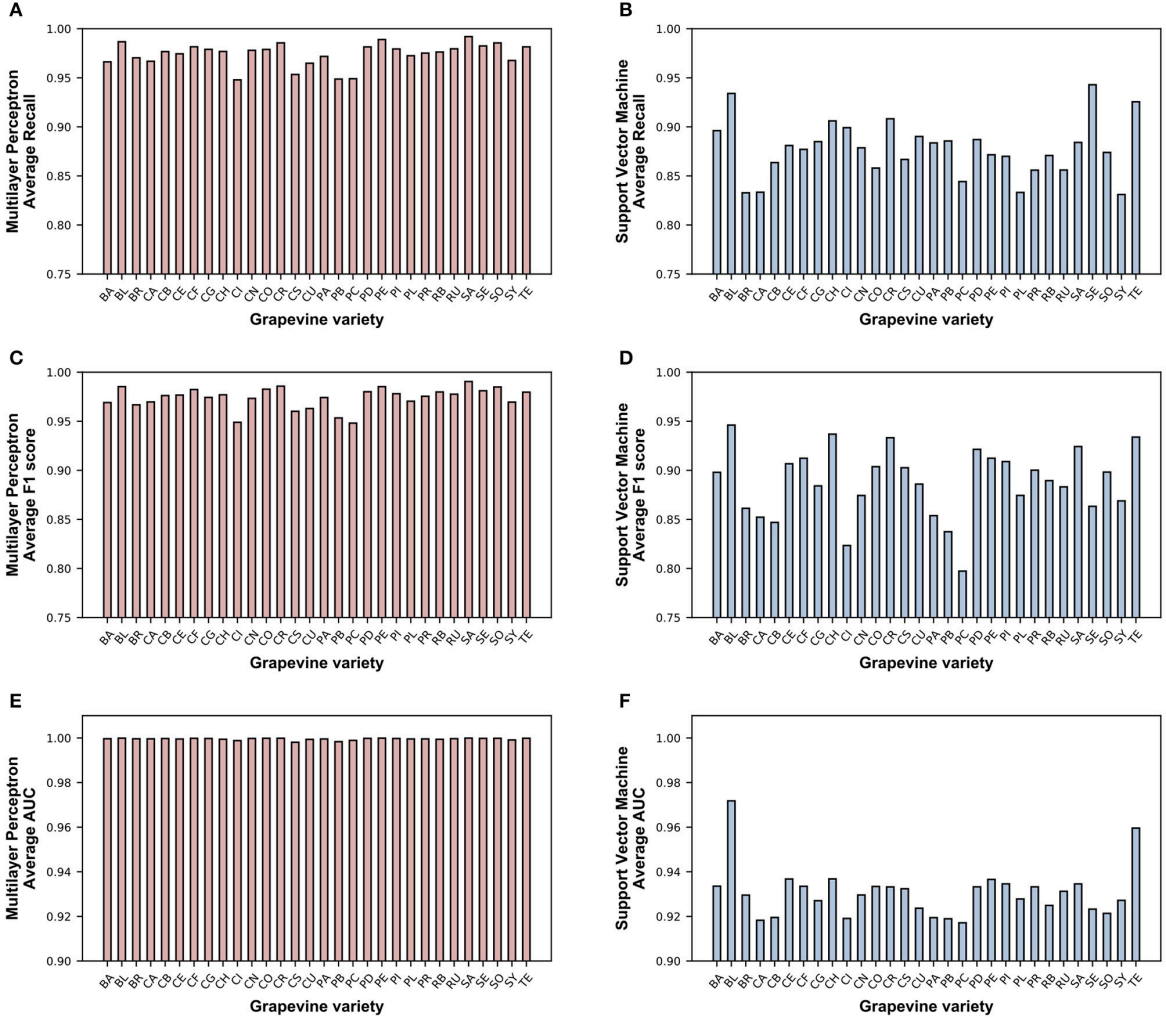
Average recall **(A,B)**, F1 score **(C,D)** and area under the receiver operating characteristic curve, AUC, **(E,F)** per grapevine variety (n = 2160) for Multilayer Perceptron **(A,C,E)** and Support Vector Machine **(B,D,F)**. BA, Baladí; BL, Blanca Cayetana; BR, Brancellao; CA, Catalán Blanco; CB, Chenin Blanc; CE, Centurion; CF, Cabernet Sauvignon; CG, Calagraño; CH, Chardonnay; CI, Cigüente; CN, Calop Negro; CO, Concord; CR, Carnelian; CS, Cabernet Franc; CU, Crujidera; PA, Palomino; PB, Pinot Blanc; PC, Picapoll Blanco; PD, Pardina; PE, Pedro Ximénez; PI, Pinot Noir; PL, Parellada; PR, Perruno Fino; RB, Rubired; RU, Rufete; SA, Sauvignon; SE, Semillón; SO, Sousón; SY, Syrah; TE, Tempranillo.

The average recall, F1 scores and AUC values, for each grapevine variety, were computed for SVM and MLP models. Figure 4 displays bar plots of these averages for the 30 varieties. No clear correlation between the trends of both algorithms was found (the ranking for best classified classes was not the same between algorithms). For recall values, the difference between the best and the worst score for MLP was 0.04, presenting a low variability, while for SVM this difference swelled to 0.11. The plot shapes between both algorithms were similar for recall (Figures 4A,B) and F1 score (Figures 4C,D), but the AUC values for MLP showed a very small variability level (Figure 4E), unlike SVM (Figure 4F).

Attending to recall values per variety, representing the ratio of correctly classified samples, the varieties that showed the best recall values for MLP were Semillón, Perruno Fino and Blanca Cayetana, while Centurión was the one with the lowest value. All grapevine varieties, still, reached or surpassed the 0.94 mark. In the case of SVM, the best scores came from Semillón and Blanca Cayetana, as in the case of MLP, and Tempranillo, in third place, with recall values of greater than 0.92. All the varieties were on or above the 0.83 mark.

### 3.5. Execution time estimation

In the processing of the hyperspectral images, the segmentation and filtering step (section 2.3.1) of the 60 images (30 varieties, two different days per variety) took approximately 27 h to complete on an Intel® Core™ i7-5820K CPU with 16 GB of RAM (with no thread optimization). This resulted in an average of 1.45 s per image column to be processed (i.e., the comparison of 900 spectra with a leaf signature and the average of the spectra marked as leaves). In the case of the prediction of an unknown spectrum by a previously MLP or SVM trained model, the time required was of 0.05 s. Therefore, the total time for obtaining an average spectra from the column of a hyperspectral image and the prediction of its variety would take 1.5 s.

## 4. Discussion

The results from the present work reveal the actual capability of on-the-go hyperspectral imaging and machine learning for the classification of grapevine varieties growing under field conditions. Two main novelties have been addressed: the successful deployment of a hyperspectral camera in the field, under uncontrolled illumination conditions, and the prediction of a very large number of classes (30). This, supported by the wide evaluation of different machine learning classifiers and parameters, made possible to obtain classification results up to 0.99 for both SVM and MLP. The models were able to cast notable prediction results from data acquired in two different phenological stages, correctly classifying leaves of different degree of development.

To the best of our knowledge, no previous studies can be found on in-field plant varietal classification neither on-the-go nor using ground-based hyperspectral imaging. Nevertheless, recent works have displayed the use of in-field portable NIR spectroscopy for the classification of grapevine varieties (Gutiérrez et al., 2015, 2016), discriminating among 20 and 10 different varieties, respectively. The reported cross validation classification results went up to 87.25 and 88.7%, remarkable values considering the high number of classes employed in the training of the models. The present study improved both the number of varieties discriminated and the classification response. The different spectroscopic device used (hyperspectral imaging vs. spectral measurement of a very reduced area) could be the key factor of these enhancements. A portable spectrophotometer is only capable of acquiring spectral signals from a reduced portion of the target (grapevine leaves, in this case), hence a lot of information is lost if the whole canopy is not monitored by the device. On the other hand, adding two spatial dimensions to spectral data greatly increases the quantity of the information acquired from the canopy, as all the intervariability (among plants) and intra-variability (within plants) is considered. Hence the prediction capability of the machine learning algorithms is expected to be increased, as they are fed with more information. Hyperspectral imaging has been previously attempted for the varietal classification of grapevine leaves and clones by Diago et al. (2013) and Fernandes et al. (2015), respectively. In these studies, the authors demonstrated the ability of this technology for the discrimination of samples from three varieties and four clones. However, these approaches, unlike the present study, needed for specific sample preparation. Moreover, imaging was conducted under laboratory conditions and only at harvest time, over leaves of different ages. Varietal classification by spectroscopy has been previously achieved in several agricultural and food applications. Maize seed discrimination attending to the variety was recently reported by Guo et al. (2017a) and Yang et al. (2017), with up to 14 varieties and using hyperspectral imaging and SVM. Artificial neural networks and SVM have also been used for this purpose in pummelo (Li et al., 2016), olive oil (Binetti et al., 2017), barley malt (Porker et al., 2017), or lotus seed (Guo et al., 2017b). All these studies had two common factors: the use of non-portable devices and the need of laboratory conditions. The present study tried to overcome these two major issues, by developing a methodology for varietal classification that is also able to be performed on-the-go, directly in the field, under uncontrolled illumination conditions, as on-the-go imaging brings the great advantage of covering large areas and thus acquiring a larger and richer amount of information from the crops.

The results obtained from the different spectral pre-processing steps allow to draw some interesting deductions. The fact that scatter correction had no influence in the results (no statistically significant differences were found when using and omitting SNV and de-trending) could suggest that the spectral information used as input for the classification algorithms suffered from no interferences of scatter. This might be explained by one of the main advantages of hyperspectral imaging: the huge amount of spectra that it provides. Each sample of the built dataset came from the average of approximately 43,000 leaf spectra, and this extreme averaging could have minimized the scatter influence. When it comes to smoothing filtering, the different treatments showed no significant differences for SVM, but they were influential for MLP. The second order derivative casted the best performance statistics for this algorithm, making these results to be in line with those concluded by Gutiérrez et al. (2015), for the same purpose. Although, as mentioned, smoothing treatments had no influence for SVM (a fact that could be explained by the higher variability in classification results from this algorithm), the trend in terms of average values remained similar to those of MLP.

In general, the models trained with SVM and MLP were able to return very high statistical values of classification (for specific parameters), highlighting that hyperspectral data (and the high amount of samples) retained enough information for both machine learning algorithms to successfully extract underlying classification rules, when providing a considerable amount of samples. The best results were found in those models that were trained with MLP (average performance values from 0.95 to 0.99), but SVM was also able to provide outcomes up to 0.99. Additionally AUC values per variety were much regular for MLP (Figure 4E) than for SVM (Figure 4F), implying that MLP had a higher capability to precisely classify from any class. As it can be concluded from Table 2, wider variability results came again from SVM, displaying high differences depending on the kernel selected and much larger gaps depending on the value of the *C* parameter. The influence of the kernel was statistically present, and the analyses promoted the *linear* kernel as clearly the best, setting aside more complex kernels. This enables to affirm that spectral information was better exploited when, in the case of SVM, linear approaches were applied. Other studies have also reported good performance of hyperspectral imaging and SVM when using linear kernels in other crops and fruits (Baranowski et al., 2015; Schmitter et al., 2017; Siedliska et al., 2017), and Hsu et al. (2003) also suggested the use of linear kernels when the number of attributes is large (as in the case of spectral information). Another consideration that can be extracted from the obtained results is that the penalty parameter *C* should be set at or above 10. *C* determines the strength of regularization of the SVM (larger values imply lower regularization, i.e., correct classification of training data is more important, and vice versa), so in the present case, the best results came when the correct classification of the samples from the dataset was maximized. This situation could lead to an overfitting scenario, in which testing samples that did not participate in the training of the model yield bad predictions. Nevertheless, the fact that all the models were tested by five replicates of 5-fold cross validation could evidence that the generalization capability of SVM with larger *C* values remained present, as in each fold 20% of the samples were not used in the training, but correctly classified. Even so, a virtual performance plateau was present at a *C* value of 10, as increasing it above that amount did not improve the classification results. The different values that MLP parameters could take presented a lower variability, and not a mean lied below the 0.95 mark. In the case of the hidden layer size, the tested values had influence in the results when using larger sizes (as in *i* or *t*), implying that the artificial neural networks were able to infer the rules for high classification reports on cross validation better with increased number of neurons in the hidden layer. The activation function parameter also showed statistical differences, making the rectified linear unit function or hyperbolic tangent function the candidates that best managed the input spectral data. On the other hand, the use warm start, attending to the outcomes, has no influence in the performance of the models. Regarding the classification performance by variety (Figure 4), it is noteworthy to mention that the average response of the algorithms did not exactly agree for each variety (except for the two varieties with greater recall values: Semillón and Blanca Cayetana). This would allow to affirm that each one of the machine learning algorithms extracted concrete classification rules, and thus the specific information carried by each variety's spectral data was addressed differently by each algorithm.

Based on the exposed results, plant phenotyping under field conditions using on-the-go hyperspectral imaging is an achievable goal in precision viticulture, and has a strong potential not only for the varietal classification task, but for the prediction of many useful parameters (e.g., water status, nutritional status, disease detection, fruit composition, etc.). The effective monitoring of the vineyard can be performed in real time and georeferenced, taking advantage of the integration between sensors and computing. Some other published works support the viability of on-the-go hyperspectral imaging (Underwood et al., 2017; Wendel and Underwood, 2017; Williams et al., 2017). The methodology exposed in the present work takes into consideration the works and machinery that are employed in the vineyard. Hyperspectral imaging was performed at 5 km/h, a speed commonly found in vineyard operations from agricultural vehicles, so the integration of a hyperspectral camera with a processing hardware could by translated into a vehicle (e.g., a tractor) to acquire and compute the spectral signals in real time. The numbers exposed in section 3.5 that the segmentation, averaging and machine learning prediction of a whole hyperspectral line (column in the image) would take 1.5 s. Considering this, a hyperspectral camera could be set up for acquiring two spectra per plant, thus taking 30 s for each 10 plants to provide the predictive output (that can also be considerably reduced with hardware and software optimization). This real-time response could be in line with the way of working in current viticulture, as a fast, on-the-go varietal classification could be an useful phenotyping tool for commercial vineyards, nurseries, appellation boards, etc. Additionally, it would be possible for this integration, among many other instruments, to be deployed in agricultural robots, as demonstrated by many works found in the literature (Ruckelshausen et al., 2009; Weiss and Biber, 2011; Cheein and Carelli, 2013; Bargoti and Underwood, 2017; Underwood et al., 2017; Wendel and Underwood, 2017). The deployment of the application described in this study is also bolstered by the use of samples from different phenological states. This brings the advantage of performing on-the-go hyperspectral imaging for varietal classification at different times of the season, due to the fact that the developed models—trained with leaves from different ages—were able to notably modeling the different phenological features from the measured leaves.

As in-field varietal classification by on-the-go hyperspectral imaging and machine learning has been successfully proven within a vineyard, it is advisable to perform additional research covering supplementary aspects. The involvement of samples from the same varieties but from different locations or seasons could contribute to a richer dataset and a deeper understanding of the relationship between the spectral signal and the variety of the plant. Finally, dimensionality reduction is an interesting research topic that could focus on the future development of cheaper multispectral devices.

## 5. Conclusions

The present study displayed the actual capability of on-the-go hyperspectral imaging under field conditions for the classification of many grapevine varieties using machine learning. The results from the models obtained from testing different algorithm parameters and spectral pre-processing techniques demonstrate that a new way is opened for the task of plant phenotyping, as hyperspectral imaging has been usually performed under laboratory conditions and restricted to a selected, relatively small amount of samples. Both support vector machines and artificial neural networks, when selecting the proper parameters, proved to be reliable modeling algorithms for the training of precise classifiers. This could let for a hyperspectral imaging system to be attached to an agricultural vehicle as a phenotyping tool for real time, on-the-go classification of grapevine varieties, bringing information very useful in the context of plant phenotyping and precision viticulture.

## Author contributions

JT and SG: conceived and designed the experiments; SG and JF-N: performed the experiments: SG and JF-N: analyzed the data: JT and MD: contributed reagents, materials, analysis tools; SG, JT, JF-N, and MD: wrote the paper.

### Conflict of interest statement

The authors declare that the research was conducted in the absence of any commercial or financial relationships that could be construed as a potential conflict of interest.
